# Population Structure Promotes the Evolution of Intuitive Cooperation and Inhibits Deliberation

**DOI:** 10.1038/s41598-018-24473-1

**Published:** 2018-04-19

**Authors:** Mohsen Mosleh, David G. Rand

**Affiliations:** 1 Department of Psychology, Yale University, New Haven, CT 06511 USA; 2 Department of Economics, Yale University, New Haven, CT 06511 USA; 3 School of Management, Yale University, New Haven, CT 06511 USA

## Abstract

Spatial structure is one of the most studied mechanisms in evolutionary game theory. Here, we explore the consequences of spatial structure for a question which has received considerable empirical and theoretical attention in recent years, but has not yet been studied from a network perspective: whether cooperation relies on intuitive predispositions or deliberative self-control. We examine this question using a model which integrates the “dual-process” framework from cognitive science with evolutionary game theory, and considers the evolution of agents who are embedded within a social network and only interact with their neighbors. In line with past work in well-mixed populations, we find that selection favors either the intuitive defector strategy which never deliberates, or the dual-process cooperator strategy which intuitively cooperates but uses deliberation to switch to defection when doing so is payoff-maximizing. We find that sparser networks (i.e., smaller average degree) facilitate the success of dual-process cooperators over intuitive defectors, while also reducing the level of deliberation that dual-process cooperators engage in; and that these results generalize across different kinds of networks. These observations demonstrate the important role that spatial structure can have not just on the evolution of cooperation, but on the co-evolution of cooperation and cognition.

## Introduction

Understanding the evolution of cooperation, which is collectively beneficial but individually costly, is a major focus of research in a wide range of fields including computer science, psychology, economics, and evolutionary biology. To that end, a great deal of work has illuminated various mechanisms which can promote the evolution of cooperative behavior^1^. In recent years, work on the evolution of cooperation has begun to consider not just cooperative or non-cooperative choices, but also the cognitive processes underlying these choices^2–4^ (for a mini-review, see ref.^5^). This work has explored cognition using the “dual-process” framework^6–9^, in which decisions are made based on two different cognitive processes: (1) Automatic, intuitive and relatively effortless yet inflexible processes; versus (2) controlled, deliberate and relatively effortful but flexible processes.

Motivating the theoretical investigation of intuition, deliberation and the evolution of cooperation is a body of empirical work using economic game experiments^10–31^. In these studies, participants make incentivized choices about paying costs to benefit others, and are experimentally induced to rely relatively more on intuition or deliberation. For example, participants may be placed under time pressure, made to complete another cognitively demanding task while making their decision, or simply asked to respond using their intuition or careful reasoning. A meta-analysis of studies examining positively non-zero-sum cooperation games of the type typically studied in evolutionary game theory models (e.g., the Prisoner’s Dilemma, PD) found that intuitive gut responses tend to support cooperation, while deliberation undermines cooperation in games where defection is strictly payoff-maximizing (e.g., 1-shot PDs) but supports cooperation in games where cooperation can pay off (e.g., repeated games)^32^. [Note that while a subsequent multi-lab pre-registered replication project raised questions about a causal effect of time pressure on cooperation in 1-shot social dilemmas^11^, 66% of participants in the time pressure condition of those experiments did not respond within the allotted time^12^, and a more recent pre-registered study solved this non-compliance problem and confirmed prior conclusions that cooperation was higher under time pressure than time delay^24^]. Similar results regarding intuitive cooperation were also found in a field experiment on real-world helping behavior^33^, and when analyzing interviews with people who risked their lives to save strangers^34^. This pattern of results was explained by a verbal theory, the Social Heuristics Hypothesis (SHH)^16,35^. The SHH postulates that typically advantageous (i.e., long-run payoff maximizing) behaviors become automatized as intuitive default responses, whereas deliberation can override these intuitive defaults to better match the strategic details of the current situation at hand.

Evolutionary game theoretic models of the co-evolution of cognition and cooperation sought to explore this question formally, conceptualizing intuition versus deliberation as a trade-off between ease and flexibility, and asking what intuitive and deliberation behaviors would be favored by natural selection^2–4^. The results have indicated that when future consequences are sufficiently likely, natural selection favors a “dual-process cooperation” strategy that accords with the experimental results: this strategy is intuitively predisposed to cooperate, but uses deliberation to overrule that predisposition and instead defect when doing so is payoff-maximizing (e.g., in 1-shot anonymous interactions).

This prior work on the co-evolution of cooperation and cognition, however, has only considered well-mixed populations. A separate (and much older) line of work examining structured populations has shown that the topology of interaction affects the evolution of cooperation^36–53^. In particular, non-random interactions can facilitate the evolution of cooperation, even allowing cooperation to succeed in 1-shot Prisoner’s Dilemma games. Experimental work has also explored the role of structure, and although some studies have found little impact of structure on cooperation^54–60^, it has been shown that structure *does* promote cooperation when particular theoretically-derived conditions are satisfied^61^.

None of this work on population structure, however, has considered the role of intuition versus deliberation. Here we bridge these two approaches to investigate the effect of interaction structure on the co-evolution of cognition and cooperation. We explore conditions (network structures and frequency of one-shot versus repeated games) under which natural selection favors costly deliberation over intuition, as well as cooperative over selfish intuitive responses. In doing so, we shed light on the role of network structure in shaping not only our actions, but also the thought processes that give rise to those actions.

To do so, we adapt a model of the co-evolution of cooperation and cognition proposed for well-mixed populations^2^. In each generation, agents play a series of Prisoner Dilemma (PD) games in which they can either choose to always defect (ALLD) or to play the reciprocal strategy tit-for-tat (TFT) which cooperates in the first period and then copies the partner’s move from the previous period. The PD games come in one of two types: with probability 1-*p* it is a one-shot anonymous game (in which defecting is strictly payoff-maximizing); whereas with probability *p* it is an infinitely repeated game (such that it is payoff-maximizing to play the same strategy as the partner). Cognition is modeled as follows. In each game, each agent can either choose her strategy using a generalized intuitive strategy that is independent of the game type; or she can pay a cost (stochastically sampled decision-by-decision) to deliberate and tailor her strategy choice to whether the game is one-shot or repeated. Thus, each agent has a strategy vector that contains the following four elements: *S*_*i*_, the probability of TFT when the agent decides intuitively and is agnostic to the game type; *S*_*1*_, the probability of TFT in one-shot PDs when the agent deliberates and tailors her strategy; *S*_*r*_, the probability of TFT in repeated PDs when the agent deliberates and tailors her strategy; and *T*, the maximum cost which the agent is willing to pay to deliberate. In each interaction, the agent’s cost of deliberation (d*) is drawn from a uniform distribution [0,1]. The cognitive processing mode (intuition versus deliberation) is then determined by the cost threshold *T*: if d* ≤ *T*, the agent pays the cost and deliberates, and if d* > *T*, the agent plays both games with the same generalized strategy *S*_*i*_.

Our key contribution is to add interaction structure to this model. We do so by specifying a network where agents are represented as nodes and only interact with their immediate neighbors, and where agents update their strategies using the death-birth process with exponential fitness. In this evolutionary dynamic, each generation every agent has a fixed strategy vector and accumulates payoffs across games with all of her neighbors; and then at the end of each generation, an agent is randomly selected to update, and her strategy is replaced with a neighbor selected proportional to an exponential function of the neighbors’ game scores (or, with probability *u*, a mutation occurs and a randomly drawn strategy is substituted instead)^62^. This process can represent either genetic evolution, in which case the updating agent dies and the replacing agent reproduces, or cultural evolution/social learning, in which case the updating agent imitates the replacing agent’s strategy. Within this model setup, we examine the influence of population structure on the coevolution of cooperation and cognition by determining the impact of varying the average number of neighbors on the evolutionary outcomes.

## Results

**Figure 1:**
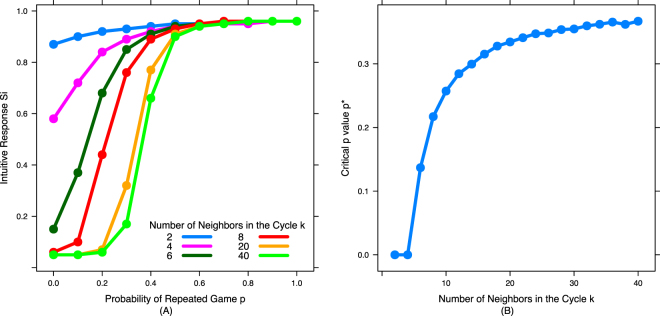
Network structure promotes the evolution of intuitive cooperation: As the density of network connections decreases, it becomes easier for selection to favor intuitive cooperators. Shown is the average intuitive response (*S*_*i*_, probability of playing TFT) across different values of *p* (probability of repeated games) for cycles with different number of neighbors (*k*) for each node. (**A**) *S*_*i*_ for six representative values of *k* across the full range of  *p*. (**B**) Critical value of *p* at which *S*_*i*_ = 0.5 across the full range of *k*. As *k* decreases, the transition from *S*_*i*_ = 0 to *S*_*i*_ = 1 occurs at lower values of *p*.

**Figure 2:**
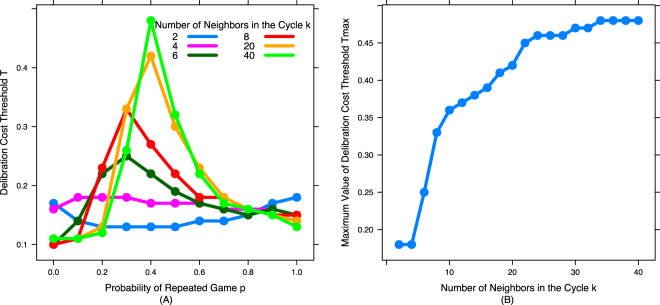
Network structure reduces the amount of deliberation: As the density of network connections decreases, the maximum cost agents are willing to pay to deliberation decreases. (**A**) Cost threshold of deliberation (*T*) for six representative values of *k* across the full range of probabilities of repeated game (*p*), (**B**) maximum value of cost threshold of deliberation over all *p* values (*T*_*max*_) as a function of number of neighbors in the cycle (*k*). As *k* decreases, dual process cooperators engage in less deliberation (*T*_*max*_ decreases).

We begin by examining the evolutionary outcomes on cycles, networks in which each agent is connected to *k*/2 neighbors on each side (for a total of *k* neighbors). In particular, we consider the average value of each strategy parameter in steady state, and ask how these values vary based on *p* (probability of repeated games) and *k* (number of neighbors). Figure 1A shows the average value of *S*_*i*_ (intuitive response) as a function of *p* and *k*. To summarize the impact of *k* on *S*_*i*_, we fit a Sigmoid function to the *S*_*i*_ curve for each value of *k* and then use that to find the critical value of *p* at which *S*_*i*_ equals 0.5 (which we refer to as p*) - representing the probability of repeated games at which the predominant intuitive strategy transitions from defection to cooperation (Fig. 1B). Figure 2A shows the value of the deliberation cost threshold *T* as a function of *p* for different values of *k*, and Fig. 2B summarizes the results by showing the maximum value of *T* over all *p*’s (*T*_*max*_) for each value of *k*.

**Figure 3:**
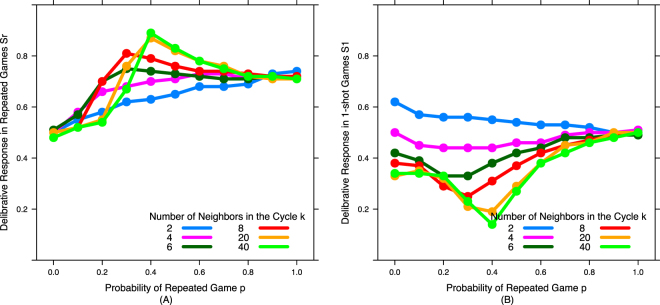
Network structure has little qualitative impact on deliberative responses in repeated games, but promotes deliberative cooperation in one-shot games. (**A**) Deliberative response in repeated games (*S*_*r*_) and (**B**) Deliberative response in 1-shot games (*S*_*1*_) across different values of probability of repeated game (*p*) for different number of neighbors in the cycle (*k*).

We see that for high values of *k* (highly connected networks) the results match those found previously in well-mixed populations^2^: For low values of *p*, intuitive defectors (ID) who never deliberate (*S*_*i*_ = 0, *T* = 0) are dominant (deliberative strategies *S*_*1*_ and *S*_*r*_ rarely used and thus dominated by neutral drifted around 0.5 - Fig. 3); but once *p* becomes sufficiently high, the dual-process cooperator (DC) strategy (*S*_*i*_ = 1, *S*_*r*_ = 1, *S*_*1*_ = 0, *T* = *c*(1-*p*)) dominates (and as *T* approaches 0, *S*_*1*_ and *S*_*r*_ are used less and less, and thus get pulled back towards 0.5 by neutral drift - Fig. 3).

**Figure 4:**
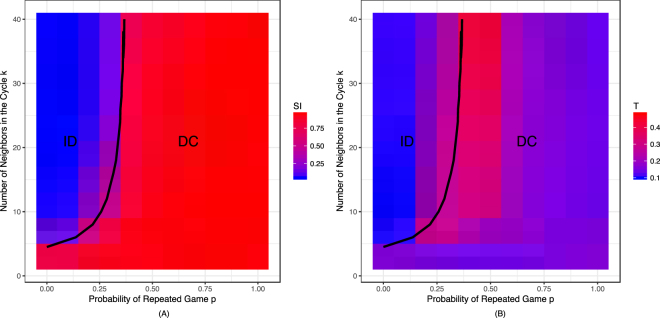
Dual-process cooperators evolve when it is sufficiently likely that games are repeated and/or the network is sufficiently sparse. (**A**) Probability of intuitive cooperation (*S*_*i*_) and (**B**) cost threshold of deliberation (*T*) as a function of number of neighbors in the cycle (*k*) and probability of repeated game (*p*). The black lines in both panels represent the value of *k* and *p* at which the dominant strategy transitions from ID to DC. There are only two dominant strategies: for high values of *k* and low values of *p* the intuitive defectors (ID) who never deliberate are dominant, while for low values of *k* and high values of *p* the dual-process cooperator (DC) strategy dominates. Lower value of *k* makes it easier for the DC strategy to succeed, and reduces the amount of deliberation DC agents are willing to engage in.

As the number of neighbors in the network (as thus density of connections) decreases, however, we observe marked impacts on both *S*_*i*_ and *T*: the emergence of the dual-process cooperator strategy (*S*_*i*_ = 1) takes place at lower values of *p*, and these dual-process cooperators engage in less deliberation (*T*_*max*_ decreases). These results are visualized more fully in Fig. 4, which shows heatmaps of *S*_*i*_ and *T* as a function of *k* and *p*. In sum, we see that as in the well-mixed population, there are only two dominant strategies: for high values of *k* and low values of *p* the intuitive defectors (ID) who never deliberate are dominant, while for low values of *k* and high values of *p* the dual-process cooperator (DC) strategy dominates. Rather than introducing new successful strategies, interaction structure makes it easier for the DC strategy to succeed, and reduces the amount of deliberation DC agents are willing to engage in.

**Figure 5:**
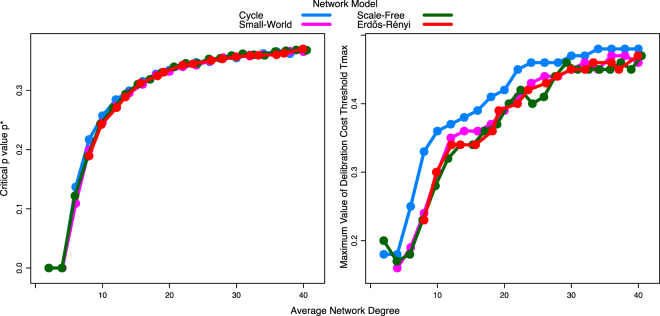
Similar evolutionary dynamics are observed across varying network structures. (**A**) Critical value of probability of repeated game *p* at which *S*_*i*_ = 0.5 and, (**B**) Maximum value of cost threshold of deliberation *T* over all values of *p*, across average network degree *k* for Cycle, Watts-Strogatz Small-World, Barabási-Albert Scale-Free, and Erdős-Rényi random networks. Results obtained for cycles are robust to networks with heterogeneous degree suggesting that the effect of structure on the coevolution of cooperation and cognition is mainly driven by the sparsity of connections within the network.

So far, we looked at the evolutionary dynamics on cycles with homogeneous structure. We now demonstrate the robustness of our results to considering various network structures that are heterogeneous (i.e., not all agents have the same number of neighbors).To do so, we generated heterogeneous networks using the following network models: Watts-Strogatz Small-World^63^, Barabási-Albert Scale-Free^64^, and Erdős-Rény^65^ random networks. Figure 5 summarizes how changing average degree *k* influences the evolutionary outcomes for each network structure. We see that p* and *T*_*ma*x_ follow an extremely similar pattern across all network structures, showing that our results are robust to heterogeneous networks. Furthermore, the extreme level of similarity suggests that the effect of structure on the coevolution of cooperation and cognition is mainly driven by the sparsity of connections within the network, rather than other properties of the network structure.

## Discussion

Our results demonstrate how network topology, and in particular sparsity of connections, can have an important impact on the co-evolution of cooperation and cognition: a small number of neighbors per agent results in higher cooperation even when repeated interactions is rare; it also increases the tendency of agents to rely more on intuitive impulses. This was true across a range of different network structures. More broadly, our results show the robustness of dual-process cooperation to relaxing the unrealistic assumption of random matching: even in structured populations, evolution only ever favors agents who (i) always intuitively defect, or (ii) are intuitively predisposed to cooperate but who, when deliberating, switch to defection if it is in their self-interest to do so. However, the specific conditions under which this transition between the two strategies occurs depends strongly on the network structure, with more spare networks allowing dual-process cooperation to dominate for lower probabilities of repeated games; and the specifics of the dual-process strategy also varying with population structure, such that more sparse networks lead to less willingness to deliberate.

Why is this so? Reducing the number of neighbors (*k*) in the cycle leads to greater assortment^66^: When agents only interact with their neighbors in a sparse network, the emergence of clusters in which agents interact with other agents who have similar strategies is facilitated. Formation of clusters helps cooperators to more likely interact with other cooperators and collect mutual benefits of cooperation. This increases cooperators’ payoffs relative to defectors and helps stabilize cooperation. Hence, decreasing *k* makes cooperation more beneficial in the 1-shot game and results in transition to DC for lower values of *p*. For very low values of *k* (*k* < *b*/*c*, where *b* and *c* are, respectively, benefit and cost of cooperation^42^) the favored strategy is always cooperation across all *p* values. This increase in assortment also reduces the value of deliberation (and thus reduces the cost that agents are willing to pay to deliberate *T*), because as the likelihood that your partner has the same strategy as you increases, it becomes less beneficial to switch to defection in 1-shot games: strategies that switch to defection will wind up interacting with other strategies who also defect. The results we present here using structured populations correspond nicely to prior work^2^ who vary assortment mathematically, without explicitly modeling population structure.

In our model, we made the simplifying assumption that the cost of deliberation was drawn from a uniform distribution, which has been shown to influence the evolutionary dynamics^3^: other distributions allow the success of a strategy which intuitively defects and uses deliberation to cooperate in repeated games. Considering the impact of network structure with alternative distributions of deliberation costs is therefore an important direction for future research. Similarly, we assumed that intuition was totally insensitive to context whereas deliberation was perfectly sensitive. As previous work has shown that allowing intuition to be somewhat sensitive to context can allow the success of a “dual-process attender” strategy which distinguishes between one-shot and repeated games even when using intuition^4^, the interaction between context-sensitive intuition and spatial structure is also an important direction for future work.

Another promising extension is to consider a case where the probability of repeated game is not the same for all agents, but it is a function of the way agents are connected through the network: In real world settings, people who are interacting within a densely connected community are more likely to be engaged in interactions that carry future consequences while people who are socially distanced from each other are less concerned about their future interactions. Hence, making the value of *p* heterogeneous as a function of agents’ local structure can be a natural future direction. It would also be informative to consider the coevolution of cooperation and cognition on dynamic networks, where there structure is not fixed but instead can evolve over time or can be altered by the agents^67^. Finally, our findings regarding the impact of interaction structure on the co-evolution of cognition and cooperation also suggest that extending models of the evolution of intuition and deliberation in anti-coordination contexts (e.g., using snowdrift games) and in non-cooperative contexts^68–70^ to include non-random interactions will be a valuable direction for future work. The topology of interaction is a key feature of our world, and has important impacts on both how we act and how we think.

## Model

Our results are produced using agent-based simulations. In our simulations, agents play with and adopt strategies of their immediate neighbors in the network with population size N = 100. In each encounter between two agents, the players can choose to either play TFT or ALLD. With probability *p* the game is a 1-shot PD with payoff matrix $$[\begin{array}{cc}R=(b-c) & S=-c\\ T=b & P=0\end{array}]$$ and with probability (1-*p*) they play an infinite repeated PD where agents play the stage-game from the 1-shot PD each period, yielding average payoff per round of $$[\begin{array}{cc}R=(b-c) & S=0\\ T=0 & P=0\end{array}]$$ where *b* = 4 and *c* = 1 in our simulations. The strategy of agent $i\in \{1,\ldots ,N\}$ is characterized by ${s}_{i}=({S}_{i}^{i},{S}_{r}^{i},{S}_{1}^{i},{T}^{i})$ specifying the behavior of the agent when she decides intuitively (TFT with probability ${S}_{i}^{i}$ independent from the game type) and when she decides deliberately (TFT with probability ${S}_{1}^{i}$ in the 1-shot game and TFT with probability ${S}_{r}^{i}$ in the repeated game). ${T}^{i}$ determines the maximum cost which the agent is willing to pay to deliberate: In each interaction, a random cost d* is drawn from uniform distribution [0,1], if d* ≤ *T*_*i*_ the agent deliberates and tailors her strategies and plays TFT in the 1-shot game with probability ${S}_{1}^{i}$ and plays TFT in the repeated game with probability ${S}_{r}^{i}$, otherwise she uses the intuitive strategy and plays TFT with the same probability ${S}_{i}^{i}$ regardless of game type. Once all agents have completed interactions with all their neighbors, payoffs are calculated and evolution occurs. We use Moran death-birth process with exponential payoff function. In each generation, an agent *i* is randomly selected to change strategy. The agent *i* adopts the strategy of another agent *j* in the neighborhood who is selected with probability $W({s}_{j}\,\to {s}_{i})=\exp (w{\pi }_{j})$ where ${{\rm{\pi }}}_{j}\,\,$is the averaged expected payoff of agent *j* by her degree and *w* is the selection intensity. With probability *u* mutation occurs and instead a random strategy is chosen. In our simulations, we used *w* = 4 for the selection intensity (strong selection) and *u* = 0.01 for mutation rate.

To generate homogenous networks, we used cycles where the number of neighbors for each node ${\rm{k}}\in [2,4,\ldots ,40]$. To generate heterogeneous network structures with varying average degree, we used the following network models: Watts-Strogatz Small-World networks where the number of neighbors for each node in the ring structure ${\rm{k}}\in [2,4,\ldots ,40]$ and rewiring probability ${{\rm{p}}}_{{\rm{rw}}}=0.2,\,\,$Barabási-Albert Scale-Free networks where the number of edges to add in each time step $m\in [1,2,\ldots ,23]$, and Erdős-Rényi random networks where probability of edge creation ${{\rm{p}}}_{{\rm{ec}}}\in [0.02,0.04,\ldots ,0.4]$. We ran the simulations for different values of *p* (discretized with resolution 0.1) on each network structure. At the beginning of each simulation run, each agent’s strategy is initialized from independent uniform distributions. Each simulation run continued over a number of generations until no more than one agent updates its strategy for a consecutive window of 10^4^ generations, as in prior work^71,72^. To enhance convergence, we considered a noise threshold of ε = 0.05 for strategies difference below which agents do not adopt a new strategy. All results are averaged over 1000 initializations. The simulations were done in parallel using the Yale computing cluster.
